# Routes to expanded carrier screening in the UK: The perspectives of professional stakeholders

**DOI:** 10.1007/s12687-026-00882-y

**Published:** 2026-04-22

**Authors:** Cathy Herbrand, Kriss Fearon, Pascal Borry, Lorraine Culley, Nicky Hudson, Zosia Miedzybrodzka, Sarah Norcross, Bronwyn Parry, Eva Van Steijvoort

**Affiliations:** 1 Centre for Reproduction Research, De Montfort University, 0.23 Edith Murphy House, Leicester, LE1 9BH UK; 2 Department of Public Health and Primary Care, Centre for Biomedical Ethics and Law, KU Leuven, Kapucijnenvoer 35/3 (box 7001), Leuven, 3000 Belgium; 3 Clinical Genetics Centre, University of Aberdeen & NHS Grampian, Ashgrove House Aberdeen Royal Infirmary Foresterhill Campus, Aberdeen, AB25 2ZA UK; 4 ANU College of Arts and Social Science, The Australian National University, Canberra, ACT 2600 Australia; 5 Progress Educational Trust, 140 Gray’s Inn Road, London, WC1X 8AX UK

## Abstract

Expanded carrier screening (ECS) is a genetic test, increasingly used before conception, to assess prospective parents’ risk of transmitting autosomal recessive and X-linked pathogenic genetic variants to future children. This paper is the first to describe the distinct coexisting routes to ECS in the UK, and to consider key professional stakeholders’ perspectives on the usefulness, advantages and challenges of these distinctive routes in a national context where ECS is almost exclusively available commercially with little regulatory oversight. Data are based on an extensive systematic search and mapping of ECS providers offering tests in the UK, and interviews with 38 stakeholders involved in the practice, provision, or governance of genomic reproductive technologies in the UK. This study identifies and describes three distinct, parallel routes through which ECS is taken up: unassisted conception, assisted conception (with couple’s own gametes), and assisted conception using donated gametes. We found that these routes served very different needs for clinicians, and identified inconsistency across the routes, with significant variations in cost, access, panel composition, and levels of support for test takers, especially in direct-to-consumer testing. These challenges are compounded by the absence of clear regulatory oversight. Findings suggest that ECS should not be treated as a single, uniform practice. Rather, its framing, implementation and perceived value must be understood in relation to the specific institutional, clinical, and social contexts in which it is situated. This contextual understanding is particularly important for informing the regulation and guidance of the distinct ECS offerings.

## Introduction

Expanded carrier screening (ECS) is a genetic screening test that can simultaneously identify an individual’s carrier status for large numbers of autosomal recessive and X-linked genetic conditions. This type of screening is increasingly used for reproductive purposes, usually before conception, to evaluate the risk of transmitting pathogenic variants to future children (Martin et al. 2015). ECS is being introduced in a growing number of countries and through a variety of routes and providers (Delatycki et al. 2020). While the use of ECS has been explored for specific purposes, e.g. as a reproductive screening tool for the general population (Van Steijvoort et al. 2020), as a factor affecting reproductive decision making for infertile couples (Franasiak et al. 2016), or within the context of gamete donation (Glenn, 2020; Payne et al. 2021), there remains a lack of published research on how ECS is offered via parallel routes in the same national context, and the specific opportunities and challenges each route presents from the perspective of professional stakeholders’ involved in the process. In addition, there is very little data on this topic in the UK context.

Most papers about professional stakeholders’ views of ECS discuss its clinical use and its general benefits for prospective parents. These include increased reproductive autonomy and choice (Henneman et al. 2016; Jämterud et al. 2021; van der Hout et al. 2017), reduced risk of pathogenic variants being passed down the generations (De Wert et al. 2014), and the avoidance of the birth of a child with severe, life-changing conditions and the subsequent impact on the family (Matar et al. 2016; van den Heuvel et al. 2023). ECS was also perceived by stakeholders as offering the advantages of not being ethnicity-based and therefore being more widely accessible (Janssens et al. 2017; van der Hout et al. 2017). Widespread use of ECS, and the shared understanding that it was usual for people to carry at least one pathogenic variant, was also seen as potentially reducing the stigma of being a carrier (van der Hout et al. 2017).

There is limited literature exploring the contexts in which ECS is used. In the work that does exist, fertility treatment was seen as an opportunity to offer ECS with professional support (Klein et al. 2024). Use of ECS can reduce the rate of termination of pregnancy for medical reasons or of having a disabled child; several authors regarded this as especially important for patients having assisted conception, due to the cost and emotional and physical effort of getting pregnant (Cho et al. 2013; Klein et al. 2024; Drettas et al. 2023). In the UK, 70% of fertility treatment is delivered through the private sector, and is largely self-funded. Where ECS is offered, it is taking place in an environment of increasing commercialisation. While there are concerns that this may result in business interests taking precedence over patient care (Ghinea et al. 2022), there is also evidence that patients are taking a more consumerist and cost-conscious approach to fertility treatment and associated add-ons (Hamper and Perotta, 2023). However, provision mainly through commercial providers raises issues such as lack of, or inconsistent support; panels that are focused on a wide array of variants, rather than including only severe, early-onset conditions; and an increase in requests for support being directed to public health services (Henneman et al. 2016).

Specific benefits were identified for the use of ECS in donor conception. The technology was viewed as a useful way to identify whether donors and recipients carried the same pathogenic variants. This avoids creating what Klein et al. describe as ‘carrier couples’ and consequently reduces the chance of genetic conditions in donor-conceived people (Evans et al. 2024). It is also perceived as a measure to minimise the risk of legal liability for clinics (Klein et al. 2024).

The literature also discusses professionals’ views of the challenges and limitations of ECS, including the difficulty in implementing ECS with the right information and support for test takers (Pasquier et al., 2023), the risk of anxiety for test takers (Hoarau et al., 2022), the lack of appropriate care pathways for couples receiving a positive result, and its high cost, which limits access to those who can afford it (Henneman et al. 2016). Healthcare workers working in fertility clinics were not always confident that they would be able to support patients with ECS, highlighting the need for genetics training (van den Heuvel et al. 2023; Porwal et al. 2025). In the context of fertility treatment with donor gametes, concerns have been raised about the potential decrease in the donor pool if donors carrying genetic variants are excluded (Callum and Isley 2016).

A key distinguishing feature between the countries where use of ECS is growing, is whether, as in the UK currently, it is only available privately, or whether it is also available as a public health screening offer. In Australia, the McKenzie’s Mission pilot study explored how to offer ECS nationally, at no cost, to all Australian couples, looking not only at clinical utility but also feasibility, panel size, access and cost (Fehlberg et al. 2023). A pilot project in the Netherlands also assessed the feasibility of delivering ECS at no cost via general practitioners (Schuurmans et al. 2019). In both studies, offering ECS as a public health screening programme was viewed as an important way to address ethical issues such as equity of access (Dive and Newson 2021). Other countries are also offering ECS more widely. Belgium does not run a population-wide program but, based on several small-scale pilot studies, now offers ECS to reproductive couples via genetics centres and also privately (Belgian Superior Health Council, 2017). In the US, where the use of ECS is widespread, it is not available through Medicare, although it is much cheaper than in Europe. In Spain, ECS is also a paid service, and it is a legal requirement to discuss the option of ECS with fertility patients. As we go on to show, in the UK, its use has developed primarily within clinical or market-driven frameworks, with limited integration of broader public health considerations, including equitable access. Questions about the level of population-wide risk-reduction for severe but rare genetic conditions and the cost-benefit of ECS mean its feasibility as a publicly-available genetic test funded through the National Health Service (NHS) is yet to be established (Rowe and Wright, 2020).

This paper is the first to describe the distinct routes to ECS that have emerged and which coexist in the UK and to consider key stakeholders’ perspectives on the usefulness, advantages, and challenges of these distinctive routes to ECS in this setting. ‘Route’ refers here both to a mode of access and a specific context in which ECS is offered. In this respect, the UK presents a particularly useful case for exploration, for while ECS is not offered via the NHS, various ECS offerings exist and operate with little regulatory oversight, no agreed medical pathway or specific strategic plan of delivery, unlike in some other countries where it has been adopted into mainstream healthcare. The UK can serve as a useful example for other countries where similar distinctive routes to ECS might exist.

Here we explore how ECS is perceived by professional stakeholders, such as clinicians, genetic counsellors, and test providers, and how it is being implemented in the UK, discussing each of the three identified routes in turn: unassisted conception, assisted conception with couples using their own gametes, and assisted conception using donated gametes.

## Materials and methods

The data discussed in this paper are drawn from a wider ESRC-funded project examining the emergence of ECS in the UK reproductive screening landscape and its social, ethical and policy implications. The analysis is based on data drawn from a mapping of ECS offerings in the UK (Herbrand et al. 2024) together with semi-structured online expert interviews (van Audenhove and Donders 2019). The mapping involved a comprehensive online search aimed at identifying genetic test companies, based anywhere in the world, that provided ECS testing in the UK or imported donor gametes with ECS test results as well as UK genetics clinics and UK fertility clinics (private or NHS). The search also sought to gather information on the characteristics of their ECS provision.

Interviews were conducted with a sample of 38 stakeholders involved in the practice, provision, or governance of genomic reproductive technologies in the UK. Patients’ perspectives are not reported in this paper, other than as reported by professionals. All participants were selected due to their professional experience with, or informed perspectives on, ECS practices and policies. We adopted a purposive iterative sampling strategy (Patton 2015), and identified prospective participants via policy documents, publications, and conferences, and by consultation with the project’s advisory group, coupled with the results of ECS mapping. To ensure a comprehensive range of perspectives, we aimed to recruit stakeholders working in three main sectors relevant to our study, i.e. policy, clinical and commercial sectors, which included individuals from different professions (e.g. clinical geneticists, fertility clinicians, policy specialists) (Table 1).

**Table 1:** Participants. 17 participants had direct experience of offering ECS via one or more routes: 12 participants worked for organisations that offered testing in unassisted conception, 12 for organisations that offered testing in assisted conception (for couples using their own gametes), and 15 for organisations that offered testing to gamete recipients, including one in the NHS (with Integrated Care Board (ICB) agreement and if the donor’s condition met NHS criteria). While not all participants had direct experience of offering ECS, they all had the expertise to provide informed opinions about its usefulness across different routes of access and contexts

	Policy	Fertility/genetic clinics	Genetic companies	Total
Genetic counsellors	1	5 (including 1 NHS)	2	8
Clinicians (including clinical geneticists)	1 (NHS)	12 (including 6 NHS)	2	16
Embryologists		2	1	3
Policy specialist	6			6
Other		2	3	5
Total	8	22	8	

An interview guide was developed with the project’s research team and advisory board, and then piloted with members of the advisory board. Interviews explored stakeholders’ understanding of, and personal position on, ECS offerings, their perceived benefits and disadvantages, their recommendations on practice and policy guidelines and when relevant, their experience of using ECS in practice. All interviews were conducted online, lasted between 30 and 60 min, and were then audio-recorded, professionally transcribed, checked by the RF and the PI. Transcripts were analysed thematically using inductive codes in NVivo (Braun et al. 2023).

### Perceived usefulness, advantages, and challenges in specific routes to ECS

ECS for reproductive purposes is available in the UK through three main routes and delivered in different – though partly overlapping – settings, with each presenting their own specificities: unassisted conception, assisted conception or assisted conception using donated gametes. Participants had contrasting views about in which context ECS was most useful, as this screening test was perceived as presenting different advantages and challenges in each of them. The following schema summarises the key features of each route, before discussing the routes in depth (Table 2).

**Table 2:** Schema of routes

Route	Unassisted conception	Assisted conception (own gametes)	Donor conception
Provider	Private fertility or genetics clinics, genetic testing companies, or add-on to over-the-counter ancestry or health genetic test	Fertility clinics, genetic testing companies, genetics clinics	Fertility and genetics clinics, genetic testing companies, donor bank
Access	Physician-mediated, fertility or genetics clinic, or direct to consumer	Ordered through clinic or private consultant	Ordered through clinic, donor bank or private consultant
Costs (range)	£120 per person upwards	£600 per person upwards to over £1500 per couple	Around £600 for match testing.
Testing approach	May test individuals, couples at the same time, or couples sequentially	Couples tested at the same time or sequentially (usually female first to identify X-linked conditions)	Recipients of donor gametes with ECS results may be offered match- testing or ECS
Genetic counselling	May be unavailable or an additional cost	May be provided by fertility clinician or in-house/outsourced genetic counsellor	May be provided through the clinic, genetic test company, donor bank, or outsourced.
Regulation or guideline on provision	Not covered by In Vitro Diagnostic (IVD) regulations. UK-based counselling inspected by Care Quality Commission.	No specific regulations	HFEA guidance refers to British Fertility Society guidance (Clarke et al., 2019) which does not cover ECS

### ECS for unassisted conception

This route describes couples planning a pregnancy who access ECS through private providers outside the context of fertility treatment. We found that ECS is not offered by the NHS. Our mapping of ECS in the UK identified that this screening test is currently available mainly via ‘physician mediated’ private providers (Chokoshvili et al. 2017) – i.e. test-takers need to get a signature from a doctor before going ahead - as well as being offered direct to consumer without medical intervention (e.g. 23andMe). Tests are available via private genetics clinics, private fertility clinics, and from some independent genetic consultants and other commercial companies (such as private ultrasound clinics). These private providers resell ECS tests from genetic test companies, such as iGenomix (which also sells ECS online to individuals; see below). By contrast, ‘direct-to-consumer’ tests, i.e. without medical supervision, are available online or over the counter, via resellers such as pharmacies. As an example of the complexity of choice for test takers, a national ultrasound company re-sells eight different kinds of couples’ ECS tests from three different genetic testing companies, ranging from £450 for four conditions, up to £1500 (panel size not described). Some health and ancestry tests integrate some carrier screening, such as 23andMe, which includes testing for 45 variants for £159. One UK fertility clinic offers a package including couple ECS testing for 500 disorders and pre- and post-test counselling, costing £1600; this is aimed at couples who plan an unassisted conception who want an ECS test, as well as fertility patients. There is little regulation of direct-to-consumer genetic tests, as the stakeholder from the Medicines and Healthcare products Regulatory Agency (MHRA) noted, enforcement of regulations on tests sent from outside the UK is very difficult.

According to the stakeholders, while the number of so called ‘self-referrals’ into testing remains low, the number of their clients interesting in being tested was certainly increasing.

I think there’s a lot of couples now who just want to come and get this testing done and make sure that they can have a healthy child. (SH14, genetic counsellor, private genetics clinic)

Private genetics clinics were more likely than fertility clinics to be the first point of contact for both prospective parents with no known genetic risk or fertility issues, and ‘at-risk couples’ because of consanguinity, ancestry background or family history.

Most stakeholders we interviewed felt that ECS could be valuable for any couple planning a pregnancy because it increases reproductive autonomy and so should be made available to any couple who wished to pursue it. While the likelihood of being a carrier couple (where both parties are carriers of the same pathogenic variant) is relatively low, a positive result was regarded as meaningful information that the couple may decide to act on.

People can decline, obviously. But it allows people to have that reproductive autonomy then, at the end of the day, and prevent births of significant disorders. Which then has, obviously, huge psychological impact for the family for the rest of their lives. (SH29, genetic counsellor, fertility clinic)

Most participants also considered that ECS could be useful to mitigate risks and/or prevent the transmission of genetic disorders; in addition, some highlighted its potential to prepare parents for the birth of a child with a genetic condition.

The advent of preimplantation genetic testing, it offers you an option. And even if you don’t want to take that option, it offers you knowledge that might influence what you want to do. (SH13, clinician, fertility clinic)

Another benefit mentioned was that ECS could also provide important reassurance for people who were prompted to take the test because they had friends or family who had a child with a genetic condition, which may have raised awareness of their own potential reproductive risk.

I find that most of them say, either that they know somebody who had a baby with a genetic condition, and that then made them aware that there’s a risk there that they didn’t really know about before. (SH10b, genetic counsellor, genetic testing company)

However, this route to ECS was also seen as presenting specific challenges and drawbacks. One of the main challenges to the wider use of ECS outside the clinic setting mentioned by participants was a lack of awareness of this screening option among prospective parents. This was attributed, in part, to the difficulty of identifying and reaching people who are planning to conceive, at a stage in their reproductive journey when ECS would be most relevant and when they might be most receptive to the idea of testing.

It’s not like the US where, you know, there’s so much awareness, and also there’s so much access to it that you could directly approach a commercial company and get tested like that. (SH21, clinician, genetic testing company)

Some participants also feared that ECS testing could, in some cases, make people with a low risk of a positive result unnecessarily anxious.

The individuals that will choose to have it are individuals who are already worried. […] They’re going to intensely read about those things that usually mean nothing for them. But they’re going to be really worried about what they mean. (SH06, clinician, fertility clinic)

Moreover, around half of participants had concerns about whether, and how, the test parameters and results, e.g. their scope and limitations, were communicated clearly to participants, especially when the access route to ECS was direct-to-consumer. If they had a result that was positive or that they simply could not interpret, participants worried that users may not have easy access to counsellors.

A lot of the online companies and the direct consumer companies will say, please see a genetic counsellor if this happens. Well, they don’t provide it themselves, they don’t employ genetic counsellors themselves. (SH05, genetic counsellor, fertility clinic)

Importantly, while ECS is not available routinely via the NHS, staff working for the NHS reported that direct-to-consumer and online tests have created new challenges for them by causing a significantly increased workload. For example, several participants mentioned that NHS genetics services were burdened by appointment requests from people with direct-to-consumer genetic test results (such as ancestry testing that included carrier testing), including from ECS. Prospective couples with a positive result would also contact them to be referred for further treatment, such as PGT-M, (preimplantation genetic testing for monogenetic disorders), where embryos could be created and then tested for carrier status, or for match tests in the context of gamete donation (see below).

This is a good thing for the NHS in a way, because it shows that people do trust the NHS when they want to have an unbiased opinion from the public service. But it is a burden we are not able to cope with. (SH12, clinical geneticist, NHS genetics clinic)

Several participants, in particular those working in the NHS, were also concerned about the quality of online ECS tests, especially their lack of accreditation, as well as that of their results.

And the robustness of the testing, anything that’s direct-to-consumer, I always worry about the transparency, and what platform have they used and how reliable are the results. (SH09, clinical geneticist, NHS genetics clinic)

Another geneticist from the NHS said that the direct-to-consumer test results she had been asked to interpret included genetic variants that were not disease-causing.

The fact that they’re reporting things which aren’t variants as variants, or things that aren’t disorders as, ‘he’s a carrier for this thing’ and the extreme difficulty of interpreting a variant interpretation with no family history, no information. (SH15, clinical geneticist, NHS genetics clinic)

This type of result was felt to lead to needless anxiety for test-takers and additional work for the genetics team, who had to re-analyse and interpret otherwise harmless results.

### ECS for couples using assisted conception with their own gametes

ECS can also be offered to couples using assisted conception with their own gametes. In the UK, our mapping shows that this type of offer is provided via a small number of private fertility clinics and some private independent genetic counsellors by referral from a fertility clinic. Fertility clinics either arranged their own counselling and support, or if they didn’t have the resources for this, outsourced it to a genetic testing company or an independent genetic counsellor. Depending on the providers, both partners may have ECS simultaneously or one partner (usually the woman, to ensure X-linked conditions were identified) tests with ECS first, and then the other has a ‘match test’ for the variant(s) identified in the partner before starting a cycle. The cost for this type of clinic-provided testing ranges from £900-£1500 and, in this setting, it is usually offered via fertility clinics or by reproductive genetic testing companies. Fertility clinics are regulated by the Human Fertilisation and Embryology Authority (HFEA), which, however, does not regulate the offer of ECS.

While ECS was not yet widely available in UK fertility clinics, some participants believed that one of the benefits of using it in this context was to avoid further challenges for prospective parents who were already vulnerable to distress from infertility and/or prolonged medical intervention, by increasing the likelihood of a healthy live birth. This was especially important in the context of assisted conception because each cycle was already both stressful and expensive.

I’ve seen people try for 10 years to have a baby, and then have a baby that’s affected with a genetic problem. And it’s huge, it’s always a tragedy […] So, the idea that maybe you can reduce that risk by doing some extended carrier screening is something that I’m intrinsically comfortable with. (SH13, clinician, fertility clinic)

One of the key perceived advantages of this route was that ECS could be easily offered and added to their treatment protocol, when couples were already in the clinic and receptive to information.

It’s kind of a window of opportunity to incorporate prevention of recessive genetic diseases. (SH19, clinician, genetic testing company)

So given that we are already applying a medical intervention […] If there is a tool that could prevent a very, very serious condition, even though the condition is probably very infrequent, so why wouldn’t you apply it. (SH20, clinician, fertility clinic)

Moreover, for couples undergoing fertility treatment who had a positive ECS result, follow-on treatments were already available in some clinics (such as Preimplantation Genetic Testing (PGT-M), which enables embryos to be tested for specific genetic conditions and the selection of non-affected embryos for implantation).

For some clinicians, offering ECS to fertility patients was therefore seen as having commercial value for fertility clinics, not only through the sale of the test itself, but also by creating additional opportunities to offer PGT-M. Conversely, another stakeholder speculated that clinics that did not offer PGT may not provide ECS to IVF couples because they could not offer follow-on treatment if there was a positive result, and the couple would need to go elsewhere for their treatment.

I just wonder, for the clinics who can’t offer PGT, are they worried that the result might mean that those patients don’t go ahead with their treatment for that particular clinic potentially. (SH10b, genetic counsellor, genetic testing company)

In another clinic, offering ECS to fertility patients was done for reputational reasons: they wanted to be able to provide any service that patients might want, even if the uptake and profitability was low.

It’s something we feel that we have to be able to offer if patients want it. Because we’ve always prided ourselves in this clinic that we can do everything. (SH13, clinician, private fertility clinic)

Participants nonetheless identified several challenges they believed limited the uptake of ECS among fertility patients. These included: a lack of communication from clinicians about the test; the urgency couples felt to proceed quickly with treatment; limited patient interest in the test; the cost of the test itself; and the potentially expensive and complex follow-up in the event of a positive result.

A first key issue identified was the lack of communication from clinicians regarding the availability of ECS. For instance, two participants working with fertility patients said they would only offer it to couples who asked or were selective about when they mentioned it.

It’s not something that we put out there just as a general conversation with IVF patients. […] People will have to explicitly ask for it to have it. The times I’ve brought it up is when I’ve seen a patient for another reason, for example, to discuss PGT-A testing, and they tell me they’re consanguineous, for example, then I would strongly recommend it. (SH29, genetic counsellor, fertility clinic)

This genetic counsellor emphasised that ECS was seen as a potential way to identify issues in couples already known to be at higher risk, for example if they were related, or being offered preimplantation genetic diagnosis for aneuploidy (PGT-A) for various circumstances such as advanced maternal age or recurrent miscarriage. Clinicians often held divergent views about who ECS would be most appropriate for. Some, for instance, considered ECS to be particularly relevant in the context of gamete donation, but not for couples using their own gametes. Others chose not to mention the test during the initial fertility consultation, believing that patients were already overwhelmed by the amount of essential information presented at that stage.

A few also expressed concerns that raising the option of ECS with fertility patients might be perceived as promoting an expensive add-on, which led them to avoid discussing it altogether.

We have to be quite cautious not to come across as […] trying to sell expensive products through people’s IVF journeys that may not be necessary. (SH29, genetic counsellor, private fertility clinic)

Some participants noted that the UK’s restrictions on advertising^1^ meant that potential test takers were often unaware of ECS and therefore unlikely to request it, unlike in the US, where clinics and pharmaceutical companies are allowed to advertise such services. Others pointed to broader differences in the regulatory environment as influencing uptake. For example, some participants reported that in Spain, clinicians are legally obliged to inform patients about available interventions that could reduce the risk of having a child with a genetic condition. As a result, there seems to be much greater awareness amongst fertility patients in Spain, along with a higher take-up of ECS, according to stakeholders familiar with the Spanish reproductive genetics context.

In addition to the reticence of clinicians to offer the test, participants reported that some fertility patients were reluctant to use ECS when offered. An explanation for this was the timing of the test and the associated wait for results. ECS was sometimes perceived as potentially delaying the start of fertility treatment, leading some patients to decline it for that reason.

They also get frustrated because genetic testing takes too long. The results take four weeks, at least, and it holds up the IVF. Because if you’re going to do it, […] you should wait to start the IVF cycle till you’ve got the results. (SH05, genetic counsellor, fertility clinic)

Another explanation, suggested by one stakeholder, was that some fertility patients did not perceive a need for ECS, since they would not have considered using it had they been conceiving without fertility treatment.

If you’re a couple conceiving naturally, most people would never think to have carrier screening. So, for people who are just going through IVF without using a donor, then they think, well, why would they need to have that test. (SH10b, genetic counsellor, genetic testing company)

The impact of a positive result was also mentioned as a potential challenge for fertility patients, since participants reported that couples were usually reluctant to use a donor, and they may not be able to afford or access costly PGT.

Finally, some stakeholders identified the significant cost of ECS as constraining couples’ ability to pursue additional IVF cycles if their budget is limited and they want to spend the money on multiple IVF cycles instead.

### ECS in fertility treatment with donated gametes

ECS is also used in the context of gamete donation to test donors and sometimes recipients. In the UK, very few clinics and gamete banks test donors with ECS^2^, although we have identified one UK clinic chain and one UK sperm bank that test all their donors. Donors are not required to agree to ECS (Elson et al., 2024). However, over 60 UK clinics, including 20 NHS clinics, import gametes from abroad (Human Fertilisation and Embryology Authority, 2025), mainly from the US, Denmark, and Spain.

When donor carrier status is available and a carrier-positive donor is selected from an overseas provider, ECS or a ‘match test’ for the specific pathogenetic variants carried by the donor may be offered to recipients. Where the donor’s results are known, match testing may be available via the recipients’ fertility clinic, or via the donor bank, at a cost of around £500–650. Match testing is also available via the NHS on very rare occasions under strict conditions (we found only one example of this).

Participants reported that an increasing number of couples and single people having gamete donation now use match tests to check if they carry the same variants as their selected donor. Participants described match testing (i.e. testing recipients only for variants carried by the donor) as their preferred practice, as opposed to full ECS testing for recipients (i.e. testing both the donor and recipient with a full panel of variants). This is due not only to the stress, delay and complications caused by full testing but also because if the recipient was found to be positive for a genetic variant that the donor had not been tested for, it would mean additional testing for the donor; alternatively the donor would need to be changed if they declined testing or could not be traced. It was therefore simpler to test the recipient for the donor’s known genetic variants rather than to go back to the bank for this information or ask the donor to be re-tested.

We’re not just offering every woman who’s using a sperm donor to have a full panel, it just feels that’s going to cause much more trouble. (SH05, genetic counsellor, fertility clinic)

Interestingly, a significant number of participants working in gamete donation saw ECS as a way to protect clinics from legal liability, should a child be born with a genetic condition that could have been avoided by ECS testing. This seemed especially significant in a context of what participants saw as the increasingly consumer-oriented attitudes of fertility patients in the UK fertility sector.

The reason the clinics who screen the donor are doing it is to minimise the risk to them legally of any negative outcome. Because they want to say we’ve screened to the nth degree, and so they’re doing it for their risk, not to benefit the patient really. (SH06, clinician, fertility clinic)

One participant working as a clinician in a national chain of fertility clinics commented that the liability risks were more important in the setting of gamete donation, so ECS was particularly useful and needed there.

It was a policy […] that, you want to go for an egg donation programme in our clinic, you need to go for testing. Because they don’t want to take any risks […] I think it’s to cover themselves for liabilities. (SH19, clinician, fertility clinic)

Some stakeholders also viewed the use of ECS, particularly in this context, as a way to mitigate genetic risks in cases where one donor’s gametes are used across multiple couples. In such scenarios, the potential for transmission is amplified, increasing the number of children who could be affected if a condition is passed on.

When you have a gamete donor obviously it’s a different situation [from ECS with couples using IVF] because the gamete donor might be matched with many different couples. (SH12, clinician, genetics clinic)

A key advantage of using ECS/match test in gamete donation was that if donor and recipient carried a matching pathogenic variant, it should be possible to change donor and find one without a matching condition.

If someone picks a donor who’s a carrier of SMA, say, we would just offer targeted testing of that relevant gene. If the result was positive in the recipient, they could then pick a different donor, and it would just involve a reanalysis of the data to look at the new gene that was relevant. (SH10a, genetic counsellor, genetic testing company)

Participants identified a number of limitations that are specific to the context of donor conception. The first one lies in the logistical challenges surrounding the implementation of the test. For example, the cross-border import of gametes from banks and countries with different testing panels and regulations meant that testing the recipient using the same panel as the donor could be difficult if gamete banks abroad used test providers that did not provide tests outside their country. Another problematic issue is that clinics need to set up and provide pre- and post-test genetic counselling. Two participants based in NHS fertility clinics said that it would be difficult to implement match testing for people using gamete donation because of the additional cost of providing the test and genetic counselling. They did not have resources to do the testing, or to provide genetic counselling. Another participant in the private sector explained that this is why her clinic chain had rejected the option of offering ECS or match testing to their patients.

It’s expensive, it opens a can of worms. And it’s another layer of information for patients, recipients, to have to navigate and understand, and who has the resources for that. (SH03, senior manager, fertility clinic)

The lack of clarity and specificity in the HFEA Code of Practice (2023) and the professional societies’ guidelines regarding the use of donors who had tested positive was also raised. The varying interpretations of the HFEA’s requirements meant that some clinicians felt themselves to be in somewhat precarious situations and that this has led to variable practices among fertility clinics.

My sperm bank versus the other sperm banks who choose to distribute and sell carrier donors, we all do it slightly differently, which makes it very confusing for the recipients and the treating physicians. If there was clarity in the regulation about what is to be done, then […] we’ll all do very similar things. Which would thus make it easier to understand for the recipients and the physicians. (SH32, senior manager, gamete bank)

One clinician also added that using a donor was challenging enough, without having to consider the possibility of having a child with a genetic condition. This is why, although his clinic offered ECS to couples having fertility treatment with their own gametes, they did not discuss match testing with patients having gamete donation.

As with couples using IVF, timing and costs were also seen as a deterrent to using ECS in this context: ECS can significantly add to already costly donor conception and also results in a delay in starting treatment, which participants reported as a key issue for some patients. One of the participants working for a genetic testing company explained the impact:

You’re delaying everything by a month in reality if they do get a positive hit. Because if you test the donor, […], you wait a month, they’re positive. You need to test the recipient, you need to wait another month. They’ve got their IVF cycle booked, they’ve got their medications planned, they booked their time off work, etc. So, there’s a bit of a rationale there. (SH28a, business manager, genetic testing company)

These different reasons were mentioned to explain why match testing was not always accepted when offered, and in some cases, why it was not offered to gamete recipients at all (Table 3).

**Table 3:** Findings summary

	Usefulness of ECS in this route	Advantages of this route for ECS offer	Challenges of this route for ECS offer
Non-assisted conception	• Increased reproductive autonomy• Risk mitigation/prevention• Preparing the parents for the birth of a child with a condition• Gives reassurance		• Difficulty to identify and reach out to potential users• Possible increased users’ anxiety• Lack of relevant information and appropriate support• Quality of the test and result
Assisted conception (using the couple’s own gametes)	• Avoid further challenges for already vulnerable couples • Commercial interests • Reputational benefits for the provider	• Easily added to IVF protocol • Possibility of follow-up treatments in case of positive result	• Clinicians’ reluctance to offer the test if perceived as non-essential or irrelevant • Concern about being seen to offer add-ons • Patients’ lack of interest in the test • Challenges resulting from a positive result • Extra cost and delay added to treatment
ECS in assisted conception using donated gametes	• Risk mitigation tool in a context where transmission is multiplied • Protects clinics from legal liability	• Possibility of changing the donor if a risk is identified	• Logistical challenges when dealing with imported tested gametes • Need to provide pre- and post-test genetic counselling • Lack of clear and specific policy guidelines about the use of a positive carrier donor • Additional challenge to discuss with recipients • Concerns about delaying the treatment • Extra cost

## Discussion

In the context of growing availability of ECS in the UK, mostly in the private sector, we offer new data and understandings of how this technology is being offered and perceived by professional stakeholders. Our findings show how ECS is embedded differently across three main routes in the UK, which we have referred to as ‘ECS for unassisted conception’, ‘ECS for assisted conception’ and ‘ECS for assisted conception using donated gametes.

Our findings support those of other authors regarding the benefits of ECS to carrier couples (whether using their own gametes, or donor conception) in increasing parental autonomy by providing parents with more information (Drettas et al. 2023; Henneman et al. 2016), thereby reducing the chance of having a child with a genetic condition and the related possibility of a termination of pregnancy for medical reasons (Birnie et al. 2021). There were seen to be challenges in providing ECS with the right support and information for test-takers and in ensuring care pathways existed for couples with a positive result. The need to pause treatment while testing was completed, and the high cost of testing in the UK, where ECS is not cheap or covered by the NHS or health insurance, were also previously reported barriers (Capalbo et al. 2024a, b). There was also general agreement that the culture around preconception health needed to change, to raise awareness of the option of preconception ECS. We discuss the specific features of each of our three ‘routes’ in turn.

While participants agreed on the potential usefulness of ECS for prospective parents (‘ECS for unassisted conception’) and supported its availability as a service, they also highlighted significant challenges in reaching this population. One key issue was the absence of a clear point of access for individuals planning a pregnancy and limited motivation to seek it out. Unlike individuals undergoing fertility treatment, who are often offered ECS within a clinical setting that may initiate a broader care pathway, prospective parents in the general population must actively search for a test provider. Moreover, the wide variety of testing options available can be complex to navigate, particularly in the absence of clear guidance. This becomes even more challenging in the event of a positive result, if no follow-up support is provided. Ensuring access to a high-quality, reliable test emerged as a key challenge in this route in the absence of a coordinated and overarching initiative in place. This appears as a key distinction between the UK and countries such as Australia which have aimed to resolve issues of equity of access and consistency of delivery by offering ECS as a national public health screening test.

In the UK, ECS is not available through the NHS, in contrast to an extensive offer of other healthcare genetic tests which are provided free alongside expert interpretation and support. Couples who are using ECS outside of a fertility clinic route, and have no family history of genetic conditions, do not always have easy access to support and information from genetic testing companies about the implications of the results. This leads test-takers to turn to the NHS for help with positive results, creating a demand on existing services and extending the waiting list at NHS genetics clinics. Nevertheless, those stakeholders who commented felt that the NHS did not have sufficient infrastructure or money to offer ECS as a universal public health screen.

In the route we describe as ‘ECS for assisted conception’, fertility treatment was perceived by some participants as a window of opportunity to offer ECS at a point when patients can receive accurate information and appropriate support, reinforcing the views of reproductive healthcare providers in other work (Klein et al. 2024). However, this route is currently available in only a limited number of fertility clinics in the UK. This is largely due to the additional resources required to implement and integrate ECS in practice. Stakeholders also highlighted the tensions which arise around the ECS offer in this particular route, both for clinicians and for patients. Couples, particularly if they are older, generally want to get on with treatment as quickly as possible. This creates a difficult balance between maximising the chance of achieving a pregnancy and addressing the potential risks of transmitting a pathogenic genetic variant. Moreover, this route was also seen as raising a tension between offering a potential additional source of income for clinics but reducing patients’ ability to afford further rounds of self-funded IVF, meaning that both clinicians and couples may be hesitant to use ECS. This tension has been observed as a feature of the commercial provision of ECS (Henneman et al. 2016) as well as the commercialisation of fertility treatment more generally (Hamper and Perotta, 2023).

In the case of ‘ECS for assisted conception using donated gametes’, participants described how clinics have been required to address the implications of ECS, mainly as a consequence of the increasing importation of donated gametes from banks that routinely screen their donors. The growing availability of genetically tested gametes has placed pressure on clinics to consider how to manage these results and whether, and how, to communicate them to patients. Moreover, considerable confusion persists around how existing regulations and guidelines should be interpreted in practice, contributing to much uncertainty regarding the use of donors identified as carriers and the implementation of match testing. While match-testing can be beneficial in mitigating the risk of transmitting genetic conditions, particularly in a context where the occurrence of such risks is heightened, as other studies report its uptake is also being driven by legal and institutional concerns (Matar et al., 2016). An increasing number of fertility clinics and gamete banks have begun offering ECS or match testing to recipients, not only as precautionary measure but also as a way to protect themselves from potential liability. While liability had been mentioned in other studies (Klein et al. 2024), it appears as a significant concern among UK professional stakeholders.

Overall, the use of ECS in the UK is marked by a lack of consistency across the routes, with significant variations in cost, access, panel composition, and the level of support for test takers. These disparities are especially marked in the context of direct-to-consumer testing, which some participants reported was being used as a substitute for reproductive genetic testing, due to its lower cost. Offering ECS as a public health screening test may significantly address these disparities, particularly relating to access and support for test takers. By investigating professional perspectives, the study contributes to a deeper understanding of the specificities of each route, serving distinct needs for clinicians and test-takers, as well as areas of commonality. These insights are relevant not only to the UK but also in other contexts where multiple routes are emerging in parallel.

These findings also demonstrate that ECS provision in the UK also differs in important and distinct ways form that offered in other countries where it is available. As van Dijke et al. (2022) pointed out, the healthcare system is one variable that accounts for varying approaches to implementing ECS. For example, in the Netherlands, which has piloted ECS, and Belgium, where ECS is available via regional genetics clinics, a significant proportion of fertility treatment is publicly funded, and patients do not need to choose between paying for ECS and paying for fertility treatment. By comparison, in the UK, most fertility treatment is offered through private clinics, and ECS is only available privately, so patients are already making financial choices on what they can afford to add to core fertility treatment cycles. Clinics’ commercial or financial motives may be more likely to influence practice regarding whether clinics offer ECS or how they manage the use of tested gametes with patients. Consequently, practices vary considerably in ways that are not related to their perceived utility for each route: ECS may be offered as a premium service with a package of support; testing and counselling may be outsourced for efficiency; gamete recipients may be asked not to select carrier donors because the clinic does not offer match tests; or ECS may not be offered if there is no perceived commercial value.

## Conclusion

Our findings provide a novel contribution to the literature by identifying three distinct routes to the use of ECS in the UK, and by analysing their specific implications, from the perspective of key stakeholders. Our findings suggest that ECS should not be treated as a single, uniform practice. Rather, its framing, implementation and perceived value must be understood in relation to the specific institutional, clinical, and social contexts in which it is situated. This contextual understanding is particularly important for informing the regulation and guidance of the distinct ECS offerings in the UK.

## Data Availability

The anonymised data that supports the findings of this study will be available from ESRC Re-Share after the PRECAS Project is complete.

## Footnotes

1 This was jointly produced in the UK in by the Advertising Standards Authority, Competition and Markets Authority and the HFEA. See CMA, 2021 in the references.

2 Genetic testing requirements for gamete donors and recipients are outlined in the HFEA Code of Practice 9.4 Guidance Note 11 v4 Donor recruitment, assessment and screening, Section T52 (i) (2023), which in turn refers to BFS guidance (Clarke et al. 2021). There are some mandatory genetic tests, such as cystic fibrosis.
